# Impaired urge-to-cough in elderly patients with aspiration pneumonia

**DOI:** 10.1186/1745-9974-4-11

**Published:** 2008-11-19

**Authors:** Shinsuke Yamanda, Satoru Ebihara, Takae Ebihara, Miyako Yamasaki, Takaaki Asamura, Masanori Asada, Kaori Une, Hiroyuki Arai

**Affiliations:** 1 Department of Geriatrics and Gerontology, Institute of Development, Aging and Cancer, Tohoku University, Seiryo-machi 4-1, Aoba-ku, Sendai 980-8575, Japan

## Abstract

**Background:**

The down-regulation of the cough reflex in patients with aspiration pneumonia can involve both cortical facilitatory pathways for cough and medullary reflex pathways. In order to study the possible involvement of the supramedullary system in the down-regulation of cough reflex, we evaluated the urge-to-cough in patients with aspiration pneumonia.

**Methods:**

Cough reflex sensitivity and the urge-to-cough to inhaled citric acid were evaluated in patients with at least a history of aspiration pneumonia and age-matched healthy elderly people. The cough reflex sensitivities were defined as the lowest concentration of citric acid that elicited two or more coughs (C_2_) and five or more coughs (C_5_). The urge-to-cough scores at the concentration of C_2 _and C_5_, and at the concentration of two times dilution of C_2 _(C_2_/2) and C_5 _(C_5_/2) were estimated for each subject.

**Results:**

Both C_2 _and C_5 _in the control subjects were significantly greater than those for patients with aspiration pneumonia. There were no significant differences in the urge-to-cough at C_2 _and C_5 _between control subjects and patients with aspiration pneumonia. However, the urge-to-cough scores at both C_2_/2 and C_5_/2 in patients with aspiration pneumonia were significantly lower than those in control subjects. The number of coughs at C_5_/2 was significantly greater in the control subjects than those in the patients with aspiration pneumonia whereas the number of coughs at C_2_/2 did not show a significant difference between the control subjects and the patients with aspiration pneumonia.

**Conclusion:**

The study suggests the involvement of supramedullary dysfunction in the etiology of aspiration pneumonia in the elderly. Therefore, restoration of the cough motivation system could be a new strategy to prevent aspiration pneumonia in the elderly.

## Background

Morbidity and mortality from aspiration pneumonia continues to be a major health problem in the elderly. A marked depression of cough reflex sensitivity is reported in elderly patients with aspiration pneumonia who show cerebral atrophy and lacunar infarction in the brain [1]. The risk of aspiration pneumonia in post-stroke patients is known to intimately correlate with the inhibition of the cough reflex [2,3].

Cough is usually referred to as a reflex defense mechanism mediated at the brainstem level, where sensory information arising from airway sensory receptors in response to an appropriate stimulus is processed by the medullary respiratory network to produce the motor pattern of cough. However, there is accumulating evidence indicating that human cough is under voluntary control and that higher centers such as the cerebral cortex or subcortical regions have an important role in both initiating and inhibiting reflexive cough [4,5]. Although the cough reflex is certainly subjected to influence originating from cortical or subcortical brain regions [6], understanding of the nature and function of such influences is still limited.

Cough is typically preceded by an awareness of an irritating stimulus and is perceived as a need to cough, termed the urge-to-cough [7]. In a capsaicin cough challenge test, the urge-to-cough occurred at a lower capsaicin concentration than that eliciting a motor cough, suggesting that the cough cognitive sensory process precedes the cough motor event [8]. A recent functional magnetic resonance imaging study revealed that the urge-to-cough was associated with activations in a variety of brain regions, including the insula cortex, anterior midcingulate cortex, primary sensory cortex, orbitofrontal cortex, supplementary motor area, and cerebellum [9]. The down-regulation of cough reflex in patients with aspiration pneumonia could be mediated by both cortical facilitatory pathways for cough and medullary reflex pathways [4]. However, there have been no studies investigating the cortical involvement of the down-regulation of cough reflex in patients with aspiration pneumonia. In order to study the possible involvement of the supramedullary system in the down-regulation of the cough reflex, we evaluated the urge-to-cough in patients with aspiration pneumonia.

## Methods

### Subjects

Cough reflex sensitivity and the urge-to-cough to inhaled citric acid were evaluated in patients with at least one history of aspiration pneumonia and age-matched healthy elderly people.

Patients were prospectively and consecutively recruited from those referred and admitted to the Geriatric Unit, Tohoku University Hospital for treatment of pneumonia from May 2007 to April 2008. Pneumonia was diagnosed by the presence of pulmonary infiltration on chest radiograph and computed tomography (CT) and according to systemic inflammation as determined according to white blood cell (WBC) count and C-reactive protein (CRP). The criteria for pneumonia were established according to the pneumonia guidelines of the Japan Respiratory Society [10]. In the current study, aspiration was defined according to the Japanese Study Group on Aspiration Pulmonary Disease as pneumonia in a patient with predisposition to aspiration because of dysphagia or swallowing disorders [11]. In our unit, all the elderly patients (> 75 years old) with pneumonia had fasted at the time of admission. When they recovered after treatment such as antibiotics drip infusion, we considered letting them start eating with their alert consciousness. We estimated their swallowing reflex before making the decision to start eating. The swallowing reflex was induced by a bolus injection of 1 ml distilled water into the pharynx through a nasal catheter (8 Fr). The subjects were unaware of the actual injection. Swallowing was identified by submental electromyographic (EMG) activity and visual observation of characteristic laryngeal movement. EMG activity was recorded from surface electrodes on the chin. The swallowing reflex was evaluated by the latency of response, timed from the injection to the onset of swallowing [12]. If the latency of swallowing reflex was > 5 seconds, we regarded the patients as suffering from impaired swallowing function, e.g. aspiration pneumonia.

During the entry period, 41 patients with pneumonia without an apparent past- and present-history of stroke were admitted to our 20 bed geriatric unit, and 34 patients (83%) were diagnosed as aspiration pneumonia. We performed simple chest X-ray in all of them. Among 34 patients, we performed chest CT scan in 30 patients. All 34 patients showed characteristic images of infiltrates compatible with aspiration pneumonia in the posterior segment of any of the lobes and/or lower lobe by simple chest X-ray and/or CT scan. Of 34 patients, 2 patients died and 3 patients eternally tracheostomized. Of 29 recovered patients, due to the difficulty of urge-to-cough estimation, we excluded patients with dementia using the mini-Mental State Examination (MMSE). Of 29 patients who recovered from aspiration pneumonia, 18 subjects with a MMSE score < 24 were excluded. Three patients with apparent paralysis were excluded. Finally, 8 patients (3 men) with aspiration pneumonia (70–88 years old) were enrolled for this study. From 6 patients among 8, we obtained brain images with non-contrast CT scan. The CT scan revealed that 2 patients had infarct in the deep region of middle cerebral artery territory, 2 patients in the superficial region (cortical or adjacent subcortical infarcts) of middle cerebral artery territory, and 1 patient in both the deep and superficial region of middle cerebral artery territory. One patient had infarct in the superficial region of the posterior cerebral artery territory. The diameters of all infarcts were within 1 cm.

Eleven age and sex-matched healthy elderly people (72–84 years old) as control subjects were recruited from the community by advertisement. None of the subjects were demented (MMSE scores > 23). All control subjects were never-smokers, and had no previous history of pneumonia and other respiratory diseases. None of the patients or controls were taking medication which might affect cough sensitivity such as antitussives, narcotics, or ACE inhibitors. A CT scan was obtained from only one control subject.

### Cough reflex sensitivity and urge-to-cough

Cough reflex and urge-to-cough was examined more than 3 months after negative conversion of C reactive protein after pneumonia had responded to antibiotics treatment (median 24 days, range 13–30). At the time of evaluation, the subjects were in a stable state until at least 3 months before. Simple standard instructions were given to each subject.

We evaluated the cough reflex sensitivities using citric acid because we had previously used this method to observe depressed cough in the elderly [1,3]. Cough reflex sensitivity to citric acid was evaluated with a tidal breathing nebulized solution delivered by an ultrasonic nebulizer (MU-32, Sharp Co. Ltd., Osaka, Japan) [5]. The nebulizer generated particles with a mean mass median diameter of 5.4 μm at an output of 2.2 ml/min. Citric acid was dissolved in saline, providing a two-fold incremental concentration from 0.7 to 360 mg/ml. Based on "cough sound", the number of cough was counted both audibly and visually by laboratory technicians who were unaware of the clinical details of the patients and the study purpose. Each subject inhaled a control solution of physiological saline followed by a progressively increasing concentration of citric acid. Increasing concentrations were inhaled until five or more coughs were elicited, and each nebulizer application was separated by a 2-min interval. The cough reflex sensitivities were estimated by both the lowest concentration of citric acid that elicited two or more coughs (C_2_) and the lowest concentration of citric acid that elicited five or more coughs (C_5_).

Immediately after the completion of each nebulizer application, the subject made an estimate of the urge-to-cough. The modified Borg scale was used to allow subjects to estimate the urge-to-cough [7]. The scale ranged from "no need to cough" (rated 0) and "maximum urge-to-cough" (rated 10). The urge-to-cough scale was placed in front of the subjects and the subject pointed at the scale number, which was recorded by the experimenter. To assess the intensity of the urge-to-cough, subjects were recommended to ignore other sensations such as dyspnea, burning, irritation, choking and smoke in the throat. Subjects were told that their sensation of an urge-to-cough could increase, decrease, or stay the same during the citric acid challenges, and that their use of the modified Borg scale should reflect this.

### Data analysis

The study protocol was approved by the local ethics committee and informed consent was obtained from all subjects. Data are expressed as mean (SD) except where specified otherwise. The Mann-Whitney *U *test or the chi-square test were used to compare patients with controls. A p value of < 0.05 was considered significant.

## Results

All 19 subjects completed the experiments without any difficulty or side effects. Among the 8 patients with aspiration pneumonia, 3 patients had a history of recurrent pneumonia (2–3 episodes). All subjects were leading an independent life. The characteristics of subjects are summarized in Table 1. There was no significant difference in gender, age and MMSE scores between the control subjects and patients with aspiration pneumonia.

**Table 1:** Comparison of characteristics between control and patients with aspiration pneumonia

	Control	Aspiration pneumonia	P-value
Number	11	8	
Male/Female	5/6	3/5	n.s.**
Age (years)	77.3 ± 6.3	79.4 ± 6.4	n.s.*
MMSE (points)	28.1 ± 1.2	26.4 ± 1.9	n.s.*
LTSR (seconds)	1.2 ± 0.5	8.3 ± 2.1	< 0.001*

Data are mean ± S.D. *P-values by the Mann-Whitney *U *test. **P-value by chi-square test. MMSE denotes mini-mental state examination. LTSR denotes the latent time of swallowing reflex. n.s. denotes not significant.

**Figure 1:**
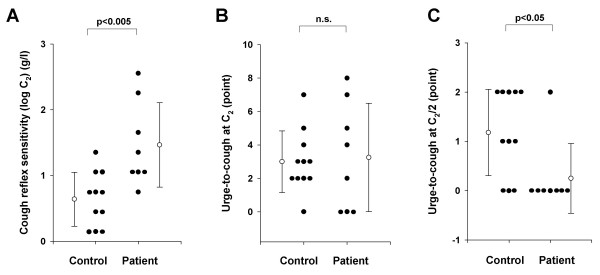
**Comparisons of cough reflex sensitivity and urge-to-cough between control subjects (Control) and patients with aspiration pneumonia (Patient).** (A) Cough reflex sensitivities expressed as the log transformation of the lowest concentration of citric acid that elicited five or more coughs (C_2_). (B) The urge-to-cough estimated by the Borg scores at C_2 _of each subject. (C) The urge-to-cough estimated by the Borg scores at the concentration of two times dilution of C_2 _(C_2_/2) of each subject. Closed circles indicate the value of each subject. Open circles and error bars indicate the mean value and the standard deviation in each group, respectively. n.s. denotes not significant.

As shown in Figure 1A, the cough reflex threshold to citric acid, as expressed by log C_2_, in patients with aspiration pneumonia (1.5 ± 0.6 g/l) was significantly higher than those of control (0.6 ± 0.4 g/l, p < 0.05). The urge-to-cough scores at the concentration of C_2 _and at the concentration of two times dilution of C_2 _(C_2_/2) were estimated for each subject. There were no significant differences in the urge-to-cough at C_2 _between control subjects (3.0 ± 1.8 points) and patients with aspiration pneumonia (3.3 ± 3.0 points) (Figure 1B). However, the urge-to-cough scores at C_2_/2 in patients with aspiration pneumonia (0.3 ± 0.7 points) were significantly lower than those in control subjects (1.2 ± 0.8 points) (Figure 1C). There was no difference in the number of coughs at C_2_/2 between the control subjects (0.1 ± 0.3 times) than in patients with aspiration pneumonia (0.0 ± 0.0 times). At C_2_/2, only one control subject coughed among all subjects.

**Figure 2:**
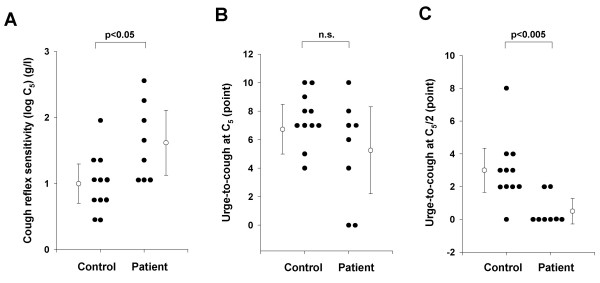
**Comparisons of cough reflex sensitivity and urge-to-cough between control subjects (Control) and patients with aspiration pneumonia (Patient).** (A) Cough reflex sensitivities expressed as the log transformation of the lowest concentration of citric acid that elicited five or more coughs (C_5_). (B) The urge-to-cough estimated by the Borg scores at C_5 _of each subject. (C) The urge-to-cough estimated by the Borg scores at the concentration of two times dilution of C_5 _(C_5_/2) of each subject. Closed circles indicate the value of each subject. Open circles and error bars indicate the mean value and the standard deviation in each group, respectively. n.s. denotes not significant.

As shown in Figure 2A, the cough reflex threshold to citric acid, as expressed by log C_5_, in patients with aspiration pneumonia (1.6 ± 0.5 g/l) was significantly higher than those of control (1.0 ± 0.4 g/l, p < 0.05). The urge-to-cough scores at the concentration of C_5 _and at the concentration of two times dilution of C_5 _(C_5_/2) were estimated for each subject. There were no significant differences in the urge-to-cough at C_5 _between control subjects (7.5 ± 1.8 points) and patients with aspiration pneumonia (5.3 ± 3.4 points) (Figure 2B). However, the urge-to-cough scores at C_5_/2 in patients with aspiration pneumonia (0.5 ± 0/9 points) were significantly lower than those in control subjects (3.0 ± 1.9 points) (Figure 2C). The number of coughs at C_5_/2 was significantly greater in the control subjects (2.3 ± 1.4 times) than in patients with aspiration pneumonia (0.75 ± 1.4 times, p < 0.05). Actually, 6 patients (75.0%) with aspiration pneumonia did not cough at all at C_5_/2 whereas 2 control subjects (18.2%) did not.

In the present study, C_2 _and C_5 _are same value in 1 subject in control group and 5 subjects in the patients with aspiration pneumonia.

## Discussion

This study shows, for the first time to our knowledge, that the urge-to-cough is significantly attenuated in elderly patients with aspiration pneumonia. It has been suggested that the aspiration pneumonia is, at least in part, a consequence of cough reflex impairment. Sekizawa and coworkers demonstrated a marked depression of the cough reflex in elderly patients with aspiration pneumonia [1]. Nakajoh and colleagues demonstrated that the greater the derangement of the cough reflex, the greater the risk of pneumonia [3]. In this study, we also showed a heightened cough reflex threshold in patients with aspiration pneumonia who did not have cognitive dysfunction and apparent paralysis. Although cough is usually referred to as a reflex controlled from the brainstem, cough can be also controlled via the higher cortical center and be related to cortical modulations. Therefore, the impairment of cough reflex could be due to the disruption of both the cortical facilitatory pathway for cough and the medullary reflex pathway. Since that the urge-to-cough is a brain component of the cough motivation-to-action system, depressed urge-to-cough suggests the impairment of supramedullary pathways of cough reflex [13].

Although we did not observe significant difference in the urge-to-cough at C_2 _and C_5_, this might be due to too small sample number in this preliminary study. However, as the urge-to-cough precedes the actual cough [7], the difference may become smaller in the point of actually coughing. This could be the reason why the difference in urge-to-cough at C_2 _was not significant between groups. Moreover, the actual cough has possibility to affect the urge-to-cough. In the study, all patients with aspiration pneumonia did not cough at C_2_/2, and 6 of 8 did not at C_5_/2. If the actual cough has ameliorating effect on the depressed urge-to-cough in the patients with aspiration pneumonia, the urge-to-cough scores at C_2 _and C_5 _became not different between groups. Well-designed and larger sample studies are warranted to clarify this.

In the present study, we estimated the cough reflex sensitivity using C_2 _and C_5_. C_5 _is considered as a clinically superior value based on better reproducibility compared to C_2 _[14]. However, Mazonne et al. assessed urge-to-cough at the concentration of C_2_/2 in order to avoid the effect of actual cough on the result [9]. In the present study, the number of coughs is significantly greater in control groups than patients with aspiration pneumonia at C_5_/2 whereas there is no significant difference in the number of cough between controls and patients with aspiration pneumonia at C_2_/2. Therefore, the urge-to-cough at C_2_/2 may more purely reflect the supramedually involvement of urge-to-cough.

Due to a lack of flow monitoring, we could not accurately distinguish between cough reflex and expiration reflex, both of which are defensive reflexes to remove foreign substances from the airway by producing the expiratory airflow. However, the latency from stimuli to induce expiration reflex was much shorter than that of cough reflex, suggesting that cortical involvement is unlikely in the expiration reflex [15]. Therefore, the urge sensation investigated here was to be the sensation for cough reflex, not for expiration reflex.

In stroke patients, an impaired cough capacity is now regarded as one of the main factors accounting for the increased prevalence of aspiration pneumonia [16-18]. The underlying mechanism of this phenomenon is still not fully understood. It is conceivable that ischemic brain damage may spread to influence the brainstem cough pathway, a phenomenon commonly referred to as 'brainstem shock'. Alternatively, it may be that ischemic brain damage of the suprameddulary area causes a loss of cortical neuro-transmission to the brainstem cough mechanism that is facilitatory to cough [19]. In this study, although our subjects did not have an obvious history of stroke, they were old enough to have silent cerebral infarction. The prevalence of silent infarction in the age group in this study was more than 15% [20,21]. Indeed, all 6 patients who had brain CT scan imaging in the present study revealed a silent cerebral infarction at various levels. A further systematic and larger sample study is required to elucidate the relationship between brain lesions and depressed urge-to-cough in the elderly.

Since it has been proposed that initiation of a reflex cough response requires the urge-to-cough to facilitate it [13], the depressed urge-to-cough could be the cause for impairment of cough reflex response in patients with aspiration pneumonia. The present study may suggest that there might be a population whose cough is impaired due to cortical or subcortical lesions rather than medullary lesions.

## Conclusion

This study suggests the involvement of supramedullary dysfunction, at least in a part, in the etiology of aspiration pneumonia in the elderly. Therefore, the restoration of the cough motivation system could be a new strategy to prevent aspiration pneumonia in the elderly.

## Abbreviations

MMSE: mini-Mental State Examination; C_2_: the lowest concentration of citric acid that elicited five or more coughs; C_2_/2: The urge-to-cough scores at the concentration of C_2 _and at the concentration of two times dilution of C_2_; C_5_: the lowest concentration of citric acid that elicited five or more coughs; C_5_/2: Urge-to-cough scores at the concentration of C_5 _and at the concentration of two times dilution of C_5_.

## Competing interests

The authors declare that they have no competing interests.

## Authors' contributions

SY, SE and TE participated in the design of the study, collected and analyzed data, and drafted the manuscript. MY, TA, MA and KU participated in the design of the study and collected the data. HA participated in design of the study and helped to draft the manuscript. All the authors read and approved the final manuscript.

## References

[B1] Sekizawa K, Ujiie Y, Itabashi S (1990). Lack of cough reflex in aspiration pneumonia. Lancet.

[B2] Addington WR, Stephens RE, Gilliland K (1999). Assessing the laryngeal cough reflex and the risk of developing pneumonia after stroke. Arch Phys Med Rehabil.

[B3] Nakajoh K, Nakagawa T, Sekizawa K (2000). Relation between incidence of pneumonia and protective reflexes in post-stroke patients with oral or tube feeding. J Intern Med.

[B4] Widdicombe J, Eccles R, Fontana G (2006). Supramedullary influences on cough. Respir Physiol Neurobiol.

[B5] Ebihara S, Saito H, Kanda A (2003). Impaired efficacy of cough in patients with Parkinson Disease. Chest.

[B6] Lee PCL, Cotterill-Jones C, Eccles R (2002). Voluntary control of cough. Pulmo Pharma Therapeutic.

[B7] Devenport PW, Sapienza CM, Bolser DC (2002). Psychophysical assessment of the urge-to-cough. Eur Respir Rev.

[B8] Davenport PW, Bolser DC, Vicroy T, Berry R, Martin AD, Hey JA, Danzig M (2007). The effect of codeine on the urge-to-cough response to inhaled capsaicin. Pulm Pharmacol Ther.

[B9] Mazzone SB, McLennan L, McGavern AE, Egan GF, Farrell MJ (2007). Representation of capsaicin-evoked urge-to-cough in the human brain using functional magnetic resonance imaging. Am J Respir Crit Care Med.

[B10] The Committee of the Japanese Respiratory Society (2002). Guidelines for management of hospital-acquired pneumonia. The basic concept of management of management of hospital-acquired pneumonia in adults [in Japanese].

[B11] Teramoto S, Fukuchi Y, Sasaki H, Sato K, Sekizawa K, Matsuse T (2008). High incidence of aspiration pneumonia in community- and hospital-acquired pneumonia in hospitalized patients: a multicenter, prospective study in Japan. J Am Geriatric Soc.

[B12] Yoshino A, Ebihara T, Ebihara S, Fuji A, Sasaki H (2001). Daily oral care and risk factors for pneumonia among nursing home patients. JAMA.

[B13] Davenport PW (2008). Urge-to-cough: What can it teach us about cough. Lung.

[B14] Dicpinigaitis PV (2007). Experimentally induced cough. Pulm Pharmacol Ther.

[B15] Tatar M, Hanacek J, Widdicombe J (2008). The expiration reflex from the trachea and bronchi. Eur Respir J.

[B16] Addington WR, Stephens RE, Gilliland KA (1999). Assessing the laryngeal cough reflex and the rsik of developing pneumoni after stroke. An interhospital comparison. Stroke.

[B17] Addington WR, Stephens RE, Gilliland KA, Rodriguez M (1999). Assesing the laryngeal cough reflex and the risk of developing pneumonia after stroke. Arch Phys Med Rehab.

[B18] Stephens RE, Addington WR, Widdicombe JG, Rekab K (2003). Effect of acute unilateral cerebral artery infarcts on voluntary cough and the laryngeal cough. Am J Phys Med Rehabil.

[B19] Stephens RE, Addington WR, Widdicombe JG (2003). Effect of acute unilateral middle cerebral artery infarct on voluntary cough and laryngeal cough reflex. Am J Phys Med Rehab.

[B20] Lee SC, Park SJ, Ki HK, Gwon HC, Chung CS, Byun HS, Shin KJ, Shin MH, Lee WR (2000). Prevalence and risk factors of silent cerebral infarction in apparently normal adults. Hypertension.

[B21] Das RR, Seshadri S, Beiser A, Kelly-Hayes M, Au R, Himali JJ, Kase CS, Benjamin EJ, Polak JF, O'Donnell CJ, Yoshita M, D'Agostino RB, DeCarli C, Wolf PA (2008). Prevalence and correlates of silent cerebral infarction in the Framingham offspring study. Stroke.

